# Relationship between mobile phone use and multidimensional frailty: evidence from UK Biobank

**DOI:** 10.3389/fpubh.2026.1868178

**Published:** 2026-08-10

**Authors:** Shuo Kong, Shuishan Xu, Songyu Wu, Changgui Kou, Wei Bai

**Affiliations:** Department of Epidemiology and Biostatistics, School of Public Health, Jilin University, Changchun, China

**Keywords:** cognitive function, longitudinal study, multidimensional frailty, older adults, physical performance, RI-CLPM, weekly mobile phone call time

## Abstract

**Objective:**

To investigate the longitudinal temporal relationships between weekly mobile phone call time and four dimensions of frailty.

**Methods:**

Data were derived from three instances (T1, T2, T3) of the UK Biobank. Frailty encompassed four dimensions: psychological, physical, social, and cognitive. Separate Random Intercept Cross-Lagged Panel Models (RI-CLPM) were constructed for each frailty dimension with weekly mobile phone call time, measured by self-reported weekly mobile phone call time standardized to baseline. Chi-square difference tests and the BIC were adopted for model comparisons.

**Results:**

This study included 5,533 participants aged 40–70 years. Unconstrained models outperformed constrained models for all dimensions (*P* < 0.001). For psychological frailty, after false discovery rate (FDR) correction, only the path from psychological frailty T1 to weekly mobile phone call time T2 was marginally significant (β = 0.086, *SE* = 0.031, *P* = 0.008, *P_FDR* = 0.048), while the other cross-lagged paths (psychological frailty T2 to weekly mobile phone call time T3: β = 0.053, *SE* = 0.023, *P* = 0.025, *P_FDR* = 0.101; weekly mobile phone call time T2 to psychological frailty T3: β = 0.067, *SE* = 0.027, *P* = 0.010, *P_FDR* = 0.069) were not significant, thus robust bidirectional associations were not supported. Physical frailty was not significantly associated with subsequent weekly mobile phone call time at either interval (T1 to T2: β = 0.035, *SE* = 0.048, *P* = 0.465; T2 to T3: β = 0.052, *SE* = 0.029, *P* = 0.075; both *P_FDR* > 0.05). Social frailty was significantly associated with subsequent weekly mobile phone call time at T2 to T3 (β = 0.078, *SE* = 0.025, *P* = 0.002, *P_FDR* = 0.033). No cross-lagged associations were observed for cognitive frailty (all *P* > 0.05). The random intercepts of weekly mobile phone call time were all significant (*P* < 0.001), suggesting stable inter-individual differences rather than within-individual fluctuations. In summary, after *FDR* correction, only two cross-lagged associations reached statistical or marginal significance: one from psychological frailty T1 to weekly mobile phone call time T2 (β = 0.086, *P*_*FDR* = 0.048), and the other from social frailty T2 to weekly mobile phone call time T3 (β = 0.078, *P*_*FDR* = 0.033), each observed in only one time interval, with no significant associations in other directions or intervals. These results indicate weak and inconsistent temporal associations rather than consistent or robust bidirectional effects.

**Conclusion:**

This study examined prospective within-person associations between weekly mobile phone call time and frailty dimensions in a middle-aged and older adult sample (baseline age 40–70 years). After FDR correction for multiple testing, most cross-lagged associations were not statistically significant. Only two paths reached significance or marginal significance: social frailty T2 → weekly mobile phone call time T3 (β = 0.078, *P_FDR* = 0.033) and psychological frailty T1 → weekly mobile phone call time T2 (β = 0.086, *P*_*FDR* = 0.048), each occurring in only one time interval. No consistent or robust bidirectional patterns were observed across dimensions or intervals. The significant random intercepts suggest stable inter-individual differences in phone use over time. Overall, these findings do not support clear temporal relationships between weekly mobile phone call time and most frailty dimensions, and the few observed associations are weak.

## 1 Introduction

Frailty is a state characterized by diminished physiological reserve and increased vulnerability, which significantly elevates the risk of adverse health outcomes (such as disability, hospitalization) and mortality in older adults following exposure to stressful events. The core issue lies in the inability of these individuals to regain the fundamental capacity to manage activities of daily living—a relatively minor illness can trigger a precipitous decline in the health status of frail individuals, sometimes even leading to death. Early research primarily focused on the physical manifestations of frailty (e.g., weight loss, exhaustion). However, contemporary perspectives have firmly established frailty as a multidimensional state of health deficits, encompassing physical, psychological, cognitive, and social functioning domains (1). Functional decline in any single dimension independently threatens the health and wellbeing of older adults. Furthermore, multidimensional frailty, as a whole, is not an inevitable consequence of aging but rather a dynamic process that may be modified through early identification and potential intervention (2, 3).

Amidst the digital wave, mobile phones have become vital tools for adults to integrate into society and access information and services, with their penetration rate within this demographic steadily increasing [the global mobile phone penetration rate among those aged 65 and above had already reached 69% in 2012 (4)]. However, the specific behavior of weekly mobile phone call time—i.e., the duration of voice calls made via mobile phones—is distinct from general smartphone use, social media engagement, online service utilization, or video calling, as it represents a real-time, synchronous voice communication mode that may have unique and domain-specific implications for health. In terms of potential benefits, regular voice calls can maintain social connectedness and provide emotional support, which may be protective against social and psychological decline. Conversely, excessive weekly mobile phone call time might contribute to physical discomfort (e.g., neck strain, hearing fatigue), psychological stress from prolonged conversations, or reduced opportunities for face-to-face social interaction, thereby potentially influencing frailty status in specific domains (5). Given these plausible yet opposing pathways, weekly mobile phone call time may exhibit differential associations with the four frailty dimensions rather than a uniform pattern across all domains.

Notably, research directly investigating the association between weekly mobile phone call time and frailty in middle-aged and older adults remains relatively limited, and existing studies possess significant limitations. Current evidence is almost exclusively cross-sectional and fails to isolate weekly mobile phone call time from broader digital behaviors or overall smartphone ownership. Current evidence is exclusively restricted to older populations, with no direct empirical research exploring the relationship between weekly mobile phone call time and frailty among middle-aged adults. For instance, while the study by Gideon Dzando et al. (6) involved weekly mobile phone call time among older adults, its focus was on analyzing the moderating role of weekly mobile phone call time on the “relationship between frailty and quality of life,” without examining the direct association between weekly mobile phone call time and frailty itself or its specific dimensions in this population. The research by Jinseon Yi et al. (7), confirmed that smartphone ownership and use were associated with a lower risk of frailty and suggested that health literacy might play a mediating role. However, this study treated frailty only as an aggregate measure, failing to elucidate the independent associations between weekly mobile phone call time and specific frailty dimensions such as physical, psychological, cognitive, and social. More importantly, both aforementioned studies employed cross-sectional designs, which precludes inference regarding the causal direction or long-term dynamic relationships between variables.

Therefore, although the relationship between weekly mobile phone call time and the health of middle-aged and older adults is receiving increasing attention, whether weekly mobile phone call time is longitudinally associated with the various dimensions of multidimensional frailty among this population still remains unclear due to a lack of large-scale prospective cohort evidence from large-scale cohort studies. To address this gap, the present study aims to utilize the three instances of follow-up data from the UK Biobank to systematically examine the longitudinal temporal associations between baseline self-reported weekly mobile phone call time and the subsequent development and progression of different frailty dimensions in adults aged 40–70 years.

## 2 Method

### 2.1 Participants

The UK Biobank is a large community-based cohort, comprising individuals aged 37 years and above across the United Kingdom (8, 9). The present study used three instances of data: T1 (Baseline/Instance 0, 2006–2010), T2 (Instance 2, 2014+), and T3 (Instance 3, 2019+). For brevity, these are referred to as T1, T2, and T3 throughout the manuscript. The UK Biobank Data from Instance 1 (2012–2013) was omitted from the present study because it lacked repeated measures of the key variable visual memory.

Sample selection adhered to the following criteria. Participants were included if they had valid weekly mobile phone call time data (UKB Field ID: 1120) at all three assessments (T1, T2, and T3). Among these, participants were excluded if, for any item across the four frailty dimensions (physical, psychological, social, and cognitive), data were missing at all three time points. The final analytical sample consisted of participants who met the WCT completeness criterion and had at least one non-missing value for every item across all four frailty dimensions (*n* = 5,533). Participants who met the WCT criterion but were excluded due to complete missingness on at least one frailty item were retained for comparison (*n* = 259; see Supplementary Table 8).

**Figure 1:**
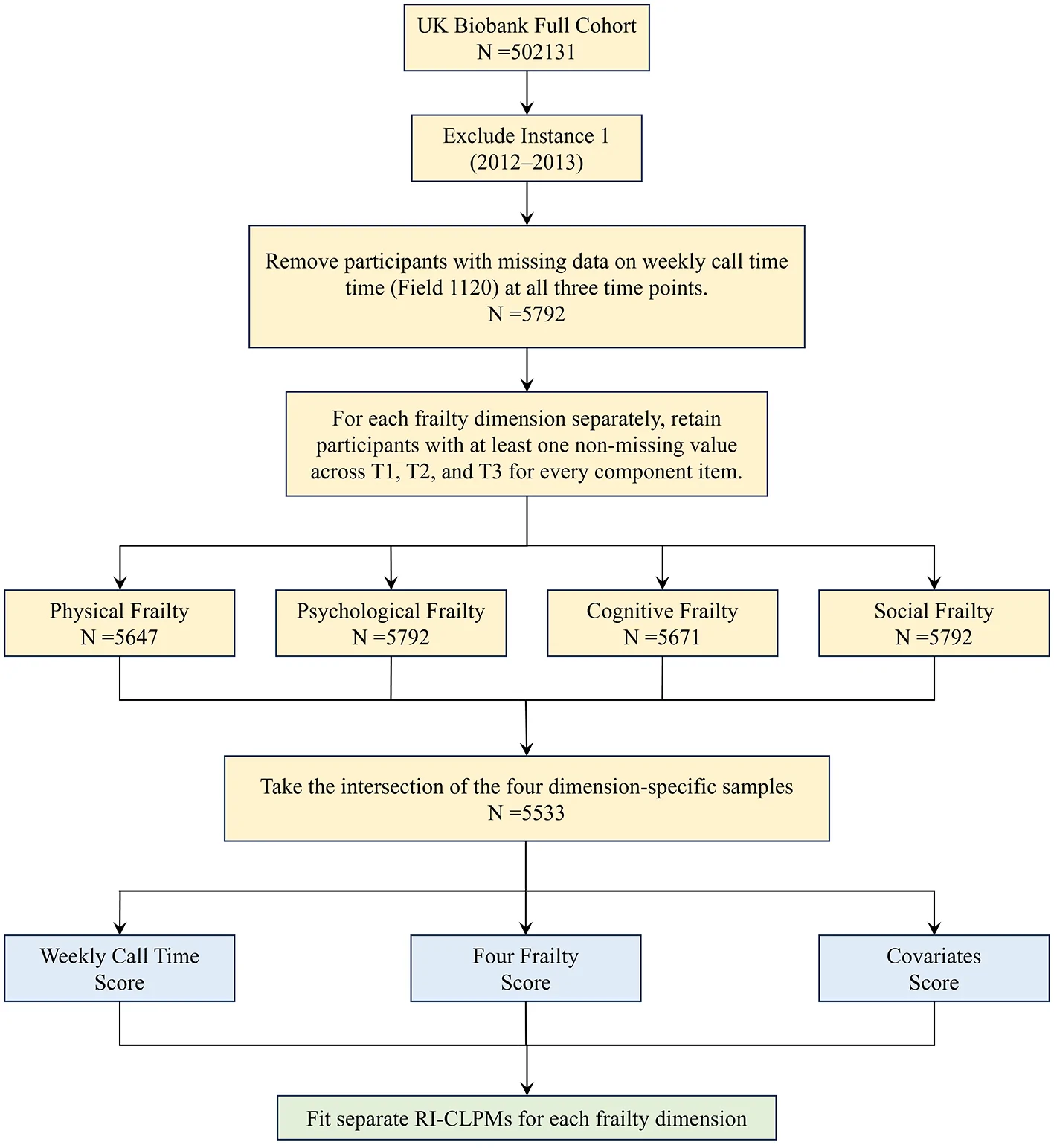
Flowchart of participant selection and analytical framework. T1, T2, and T3 correspond to the three assessment instances of the UK Biobank. T1 = baseline assessment (Instance 0, 2006–2010); T2 = first follow-up (Instance 2, 2014 onwards); T3 = second follow-up (Instance 3, 2019 onwards). WCT, weekly mobile phone call time score; RI-CLPM, random intercept cross-lagged panel model.

We used the intersection of the four dimension-specific samples as the final analytical sample to ensure comparability of results across frailty dimensions (Figure 1). If each model were fitted in a different sample, differences in estimates across dimensions could be attributable to differences in sample composition rather than true dimensional differences. Using a common sample avoids this issue and allows direct comparison of temporal associations across physical, psychological, cognitive, and social frailty.

The actual intervals between waves varied across participants: the median (IQR) was 9.03 (8.17–9.89) years from T1 to T2, and 2.37 (1.20–3.97) years from T2 to T3, with ranges of 4.41–12.70 and 1.04–7.34 years, respectively. Although described as approximately 4–5 years throughout the manuscript for brevity, there was considerable variability in the actual follow-up time across participants.

### 2.2 Measures

#### 2.2.1 Frailty

Frailty was conceptualized as a continuous variable encompassing four dimensions: physical, psychological, cognitive, and social frailty. Following the deficit accumulation model (10), each component item was assigned a value between 0 and 1 (0 = no deficit, 1 = most severe), and composite scores were generated for each dimension.

For each frailty dimension, we adopted a mean-score method rather than summing raw item scores. Specifically, for each participant at each instance, the scores of all non-missing items within a given dimension were summed and then divided by the number of non-missing items. To ensure score reliability, a minimum completion threshold was applied: at least 9 of 11 items for physical frailty, at least 5 of 7 for psychological frailty, at least 3 of 4 for cognitive frailty, and at least 5 of 7 for social frailty were required. If the number of non-missing items fell below this threshold, the composite score for that instance was set to missing. This approach avoids discarding an entire instance due to occasional item-level missingness, thereby maximizing the retention of available information.

Physical frailty comprised 11 items (score range 0–11), psychological frailty 7 items (0–7), and social frailty 7 items (0–7); for these three dimensions, higher scores indicate greater frailty. Cognitive frailty was assessed using four cognitive tests (reaction time, fluid intelligence/reasoning, visual memory, and numeric memory); raw scores were converted to z-scores and averaged, yielding a continuous composite with no fixed range, where higher scores indicate better cognitive function (i.e., lower frailty) (11). Detailed item selection, coding, and computational procedures are provided in the Supplementary Table 2 (1). The four-domain frailty framework was adapted from previous multidimensional approaches (1). Physical frailty items were based on the Fried phenotype (12). Psychological frailty was operationalized in accordance with the Tilburg Frailty Indicator (13). Cognitive frailty was defined according to international consensus criteria (14). Social frailty items were selected following a systematic review of social frailty components (15).

#### 2.2.2 Weekly mobile phone call time

A Weekly mobile phone call time Score was constructed to longitudinally quantify an individual's weekly mobile phone call time behavior as a continuous variable. This score was calculated separately at three time points: baseline (T1, 2006–2010), T2 (2014+), and T3 (2019+).

The score was derived from a single self-reported item: average weekly mobile phone call time in last 3 months (UKB Field ID: 1120). This item reflects current usage intensity rather than cumulative usage history. The ordered categorical responses were converted into numerical scores as follows: “Less than 5 mins” = 1, “5–29 mins” = 2, “30–59 mins” = 3, “1–3 h” = 4, “4–6 h” = 5, “More than 6 h” = 6. Responses of “Do not know” or “Prefer not to answer” were coded as missing. The categorization and coding details for weekly mobile phone call time are presented in Supplementary Table 3.

To enable meaningful comparisons across instances while avoiding the confounding effect of naturally increasing usage history (e.g., years of mobile phone ownership), the numerical scores at each time point were standardized using the baseline (Instance 0) mean and standard deviation. Specifically, for each participant at each Instance [i.e., Instance 0 (T1), Instance 2 (T2), and Instance 3 (T3)], the standardized score was calculated as:


$$\begin{array}{l} PH_{instance}=\frac{{phone\text{\_}time}_{instance}-\mu_{baseline}}{\sigma_{baseline}} \end{array}$$

where μ_*baseline*_ and σ_*baseline*_ are the mean and standard deviation of the numerical scores at baseline. The resulting variables *PH*_0_, *PH*_2_, and *PH*_3_ represent weekly mobile phone call time intensity relative to baseline. Higher values indicate greater weekly mobile phone call time compared to the baseline average (16). A previous version of the weekly mobile phone call time score also included years of usage (Field ID: 1110), but sensitivity analyses indicated that the cumulative nature of that variable introduced time-related bias; therefore, only weekly mobile phone call time was retained in the primary analyses.

Although the six response categories are not equally spaced, we treated them as a continuous score following previous UK Biobank studies (16) to facilitate interpretation of standardized coefficients in the RI-CLPM framework. To address the potential concern regarding unequal spacing, we conducted a sensitivity analysis using category midpoints (in minutes per week), which yielded consistent results (Supplementary Table 9).

#### 2.2.3 Covariates

Time-invariant covariates included age (continuous), sex (male = 0, female = 1), Townsend Deprivation Index (continuous), ethnicity (White British = reference), baseline disease status (1 = presence of at least one of the top five UK Biobank conditions: hypertension, high cholesterol, asthma, osteoarthritis, or hay fever/allergic rhinitis; 0 = otherwise), baseline mental health, and employment/retirement (0 = employment, 1 = retirement). Time-varying covariates (alcohol consumption, smoking status, BMI, living arrangement, social network size, hearing/vision impairment) were adjusted at each instance using baseline values for T2 outcomes and T2 values for T3 outcomes. For details, see DAG (Supplemental Figure 2).

### 2.3 Data analyses

All analyses were performed in RStudio using the *lavaan* package. Model parameters were estimated using robust maximum likelihood (MLR). Descriptive statistics and bivariate correlations between the main variables and potential time-invariant covariates (age, sex, race, Townsend deprivation index, baseline disease status) and time-variant covariates (body mass index, employment status, alcohol consumption, smoking status) were calculated. Bivariate correlations among all key variables are shown in Supplementary Table 4. Means, standard deviations, ranges, and quartiles were computed for all primary variables.

Separate Random Intercept Cross-Lagged Panel Model (RI-CLPM) analyses were performed to infer the temporal relationship between weekly mobile phone call time and each frailty dimension across three instances. The RI-CLPM was chosen over the traditional cross-lagged panel model as the latter assumes relationships are identical across all individuals and that there are no individual differences in starting levels. The RI-CLPM is more appropriate as it estimates random intercepts for each individual, thereby decomposing the observed variance into stable, between-person differences and temporal, within-person dynamics (17). This decomposition is critical when the primary interest lies in within-person temporal dynamics, as it avoids conflating stable individual differences with intraindividual change. The RI-CLPM approach has been increasingly adopted in longitudinal studies examining dynamic associations between multidimensional frailty and health-related behaviors (1).

**Figure 2:**
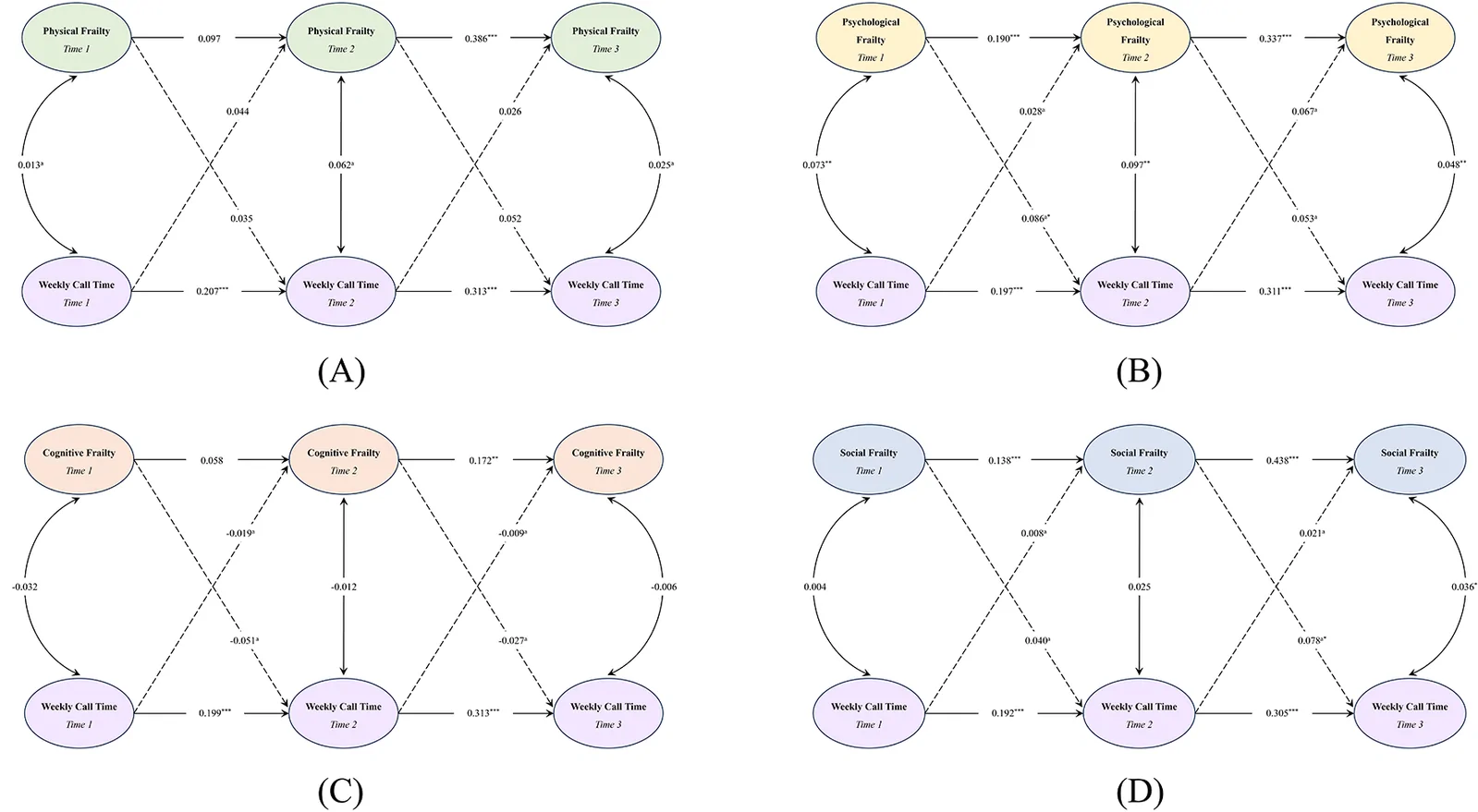
Simplified representations of RI-CLPMs for weekly mobile phone call time and each frailty dimension across three time points. **(A)** Physical frailty, **(B)** Psychological frailty, **(C)** Cognitive frailty, **(D)** Social frailty. Horizontal arrows represent autoregressive effects; diagonal dashed arrows represent cross-lagged effects; curved arrows represent within-time correlations (bivariate correlations). Standardized coefficients (β) are shown. Significance levels: *P < 0.05, ** *P* < 0.01, *** *P* < 0.001. Non-significant paths (*P* ≥ 0.05) are shown without asterisks. RI, random intercept; PH, weekly mobile phone call time. For cognitive frailty, higher scores indicate better cognitive function (i.e., lower frailty).

In the RI-CLPM, each observed variable at a given time point was decomposed into a latent random intercept (capturing trait-like, between-person stability) and a latent within-person component (representing temporal deviations from the individual's expected score). Autoregressive paths reflected the within-person carry-over stability of each construct, while cross-lagged paths indexed the prospective within-person associations from one construct to the other. Within-time residual covariances between the latent within-person components were freely estimated at each instance, and the random intercepts of the two constructs were allowed to covary (Figure 2).

Equality constraints over time were placed on the autoregressive parameters, cross-lagged parameters, and within-time residual (co)variances. The constrained model was compared against an unconstrained baseline using the Bayesian information criterion (BIC), with a lower BIC indicating better fit. A chi-square difference test was also used to compare the models. Equality tests were performed on unstandardised coefficients, with results reported as standardized coefficients (β) for interpretability. Model fit was further evaluated using the root mean square error of approximation (RMSEA < 0.05), comparative fit index (CFI > 0.95), and standardized root mean square residual (SRMR < 0.05) (18).

It is important to note that body mass index was one of the component indicators used to calculate the physical frailty score in this study. Consequently, in the RI-CLPM for physical frailty, its effect is already captured within the frailty score itself, and it was not additionally included as a separate covariate in this specific model. For the models analyzing psychological, cognitive, and social frailty, body mass index was adjusted for as a time-variant covariate. The detailed parameter estimates for all covariates in each RI-CLPM are reported in Supplementary Table 6. The contributions of these covariates to the observed outcome variables were estimated at each instance. For both covariates and the main study variables (weekly mobile phone call time and the four frailty dimensions), missing data were uniformly handled using the full information maximum likelihood (FIML) method, allowing for robust and accurate parameter estimation even when data are missing at random (19).

All autoregressive and cross-lagged effects were reported as standardized regression coefficients (β) with robust standard errors and two-tailed *p*-values based on the MLR estimator. Statistical significance was set at α = 0.05. Where the constrained model was preferred, a single estimate was reported for each constrained path; otherwise, separate estimates were presented for each time interval.

## 3 Results

### 3.1 Participant characteristics

The final analytical sample comprised 5,533 individuals aged 40–70 years at baseline (*M* = 52.50, *SD* = 7.2). Of these, 48.82% were male (*n* = 2,701) and 51.18% were female (*n* = 2,832).

Regarding health-related behaviors, 20.57% (*n* = 1,138) consumed alcohol daily or almost daily at baseline, and 5.78% (*n* = 320) were current smokers. The mean body mass index (BMI) was 26.59 kg/m^2^ (*SD* = 4.10). As shown in Supplementary Table 1, alcohol consumption, smoking status, and BMI were also assessed at subsequent instances (T2 and T3), with gradual decreases in daily drinking and smoking over time, and a slight increase in mean BMI. All demographic and health characteristics at each instance are presented in full in Supplementary Table 1.

### 3.2 WCT–frailty temporal dynamics

To determine whether the temporal dynamics between weekly mobile phone call time and each frailty dimension varied across instances, we compared an unconstrained model (all autoregressive and cross-lagged paths freely estimated) against a constrained model (paths constrained equal across intervals) using chi-square difference tests and the Bayesian Information Criterion (BIC). For all four frailty dimensions, the unconstrained model fitted significantly better than the constrained model: cognitive frailty, Δχ^2^(8) = 93.27, *P* < 0.001; physical frailty, Δχ^2^(8) = 120.52, *P* < 0.001; social frailty, Δχ^2^(8) = 149.98, *P* < 0.001; psychological frailty, Δχ^2^(8) = 282.62, *P* < 0.001. The BIC values were also consistently lower for the unconstrained models (see Supplementary Table 5). Therefore, path coefficients were allowed to vary over time, and all subsequent results are based on the unconstrained models.

Fit indices for the unconstrained models indicated mixed fit across dimensions: cognitive frailty (χ^2^(43) = 412.29, CFI = 0.939, TLI = 0.860, RMSEA = 0.041, SRMR = 0.030); physical frailty (χ^2^(31) = 377.78, CFI = 0.954, TLI = 0.880, RMSEA = 0.045, SRMR = 0.030); social frailty (χ^2^(43) = 546.80, CFI = 0.940, TLI = 0.861, RMSEA = 0.048, SRMR = 0.041); psychological frailty (χ^2^(43) = 463.54, CFI = 0.965, TLI = 0.920, RMSEA = 0.044, SRMR = 0.034). Although the CFI and RMSEA values were acceptable for most models, the TLI values for cognitive, physical, and social frailty models fell below the conventional 0.90 threshold, indicating that the model fit was suboptimal for these dimensions. The psychological frailty model showed acceptable fit across all indices. These mixed fit indices suggest that the RI-CLPM framework may not fully capture the temporal dynamics for all frailty dimensions, and the results should be interpreted with this limitation in mind (18).

#### 3.2.1 Lagged (Autoregressive) effects

In the following results, T1, T2, and T3 correspond to the three assessment instances (T1/T2/T3), with approximately 4–5 years between instances.

As presented in Table 1, weekly mobile phone call time exhibited significant positive autoregressive effects across all intervals and all models. For the physical frailty model, the standardized coefficients were β = 0.207 (*SE* = 0.030) for T1 → T2 and β = 0.313 (*SE* = 0.027) for T2 → T3 (both *P* < 0.001). Similar patterns were observed for the psychological (T1 → T2: β = 0.199, *SE* = 0.030; T2 → T3: β = 0.313, *SE* = 0.027), cognitive (T1 → T2: β = 0.197, *SE* = 0.030; T2 → T3: β = 0.311, *SE* = 0.027), and social frailty models (T1 → T2: β = 0.192, *SE* = 0.030; T2 → T3: β = 0.305, *SE* = 0.027; all *P* < 0.001).

**Table 1:** Summary of the main parameters for each RI-CLPM model – Weekly mobile phone call time and frailty dimensions across three instances (*N* = 5,533).

Parameter	Physical frailty and WCT					Psychological frailty and WCT					Cognitive frailty and WCT					Social frailty and WCT				
	β	*95% CI*	*SE*	*P*	*P_FDR*	β	*95% CI*	*SE*	*P*	*P_FDR*	β	*95% CI*	*SE*	*P*	*P_FDR*	β	*95% CI*	*SE*	*P*	*P_FDR*
Lagged (autoregressive) effects																				
Frailty T1 → Frailty T2	0.097	[−0.040, 0.235]	0.070	0.166		0.190	[0.116, 0.264]	0.038	< 0.001		0.058	[−0.078, 0.194]	0.069	0.401		0.138	[0.060, 0.216]	0.040	< 0.001	
Frailty T2 → Frailty T3	0.386	[0.312, 0.460]	0.038	< 0.001		0.337	[0.267, 0.407]	0.036	< 0.001		0.172	[0.064, 0.280]	0.055	0.002		0.438	[0.381, 0.495]	0.029	< 0.001	
WCT T1 → WCT T2	0.207	[0.148, 0.266]	0.030	< 0.001		0.197	[0.138, 0.256]	0.030	< 0.001		0.199	[0.140, 0.258]	0.030	< 0.001		0.192	[0.133, 0.251]	0.030	< 0.001	
WCT T2 → WCT T3	0.313	[0.260, 0.366]	0.027	< 0.001		0.311	[0.258, 0.364]	0.027	< 0.001		0.313	[0.260, 0.366]	0.027	< 0.001		0.305	[0.252, 0.358]	0.027	< 0.001	
Cross-lagged effects																				
WCT T1 → Frailty T2	0.044	[−0.014, 0.102]	0.029	0.134	0.357	0.028	[−0.019, 0.075]	0.024	0.246	0.394	−0.019	[−0.074, 0.036]	0.028	0.503	0.574	0.008	[−0.042, 0.058]	0.025	0.764	0.764
WCT T2 → Frailty T3	0.026	[−0.011, 0.064]	0.019	0.170	0.367	0.067	[0.014, 0.120]	0.027	0.010	0.069	−0.009	[−0.062, 0.044]	0.027	0.728	0.764	0.021	[−0.016, 0.058]	0.019	0.285	0.415
Frailty T1 → WCT T2	0.035	[−0.059, 0.129]	0.048	0.465	0.572	0.086	[0.025, 0.147]	0.031	0.008	0.048	−0.051	[−0.135, 0.033]	0.043	0.231	0.394	0.040	[−0.019, 0.099]	0.030	0.184	0.367
Frailty T2 → WCT T3	0.052	[−0.005, 0.109]	0.029	0.075	0.239	0.053	[0.006, 0.100]	0.023	0.025	0.101	−0.027	[−0.081, 0.028]	0.028	0.339	0.452	0.078	[0.029, 0.127]	0.025	0.002	0.033
Random intercepts																				
Frailty	0.449		0.032	< 0.001		0.571		0.031	< 0.001		0.462		0.032	< 0.001		0.388		0.019	< 0.001	
WCT	0.290		0.020	< 0.001		0.284		0.020	< 0.001		0.285		0.020	< 0.001		0.292		0.021	< 0.001	
Covariances																				
Frailty (kappa) → WCT (omega)	−0.041		0.050	0.414		−0.018		0.034	0.601		0.060		0.045	0.181		0.031		0.041	0.442	
Frailty T1 → WCT T1	0.013		0.039	0.748		0.073		0.025	0.004		−0.032		0.039	0.404		0.004		0.026	0.893	
Frailty T2 → WCT T2	0.062		0.033	0.060		0.097		0.030	0.001		−0.012		0.033	0.720		0.025		0.029	0.394	
Frailty T3 → WCT T3	0.025		0.018	0.174		0.048		0.021	0.021		0.006		0.023	0.799		0.036		0.018	0.046	

β represents standardized regression coefficients. For cognitive frailty only, higher scores indicate better cognitive function (i.e., lower frailty). Thus, a positive coefficient from weekly mobile phone call time to cognitive frailty would indicate that weekly mobile phone call time predicts better cognition. RI-CLPM, random-intercept cross-lagged panel model; SE, standard error. T1, T2, T3 correspond to the three assessment instances (2006–2010, 2014+, 2019+), respectively. Random intercept values represent standardized variances (i.e., proportion of total variance attributable to stable between-person differences).

The autoregressive effects of the frailty dimensions were also significant in most cases, with the exception of cognitive frailty at the T1 → T2 interval. Specifically: physical frailty (T1 → T2: β = 0.097, *SE* = 0.070, *P* = 0.166; T2 → T3: β = 0.386, *SE* = 0.038, *P* < 0.001); psychological frailty (T1 → T2: β = 0.190, *SE* = 0.038, *P* < 0.001; T2 → T3: β = 0.337, *SE* = 0.036, *P* < 0.001); cognitive frailty (T1 → T2: β = 0.058, *SE* = 0.069, *P* = 0.401; T2 → T3: β = 0.172, *SE* = 0.055, *P* = 0.002); social frailty (T1 → T2: β = 0.138, *SE* = 0.040, *P* < 0.001; T2 → T3: β = 0.438, *SE* = 0.029, *P* < 0.001). Physical and social frailty showed the strongest autoregressive effects, indicating high trait-like stability, whereas the non-significant T1 → T2 path for cognitive frailty suggests that its temporal dynamics may be more state-dependent.

#### 3.2.2 Cross-lagged effects

e × 4 cross-lagged paths per model = 16 independent tests), with an FDR threshold of q = 0.05. This family of hypotheses was restricted to the cross-lagged effects examining bidirectional temporal associations between weekly mobile phone call time and the four frailty dimensions; autoregressive paths and random intercept covariances were not included in the FDR correction. Under this procedure, the expected number of false positives (q × m) was 0.8 out of 16 tests, while the observed number of significant findings was 2.

Summary of cross-lagged findings. Cross-lagged paths from T1 to T2 were generally non-significant, with one marginal exception: psychological frailty T1 was associated with weekly mobile phone call time T2 (β = 0.086, *SE* = 0.031, *P* = 0.008, *P_FDR* = 0.048). The path from physical frailty T1 to weekly mobile phone call time T2 was not significant (β = 0.035, *SE* = 0.048, *P* = 0.465). During the T2 → T3 interval, after FDR correction, only one cross-lagged effect remained significant after FDR correction: social frailty at T2 was positively associated with subsequent weekly mobile phone call time at T3 (β = 0.078, *SE* = 0.025, *P* = 0.002, *P_FDR* = 0.033). No other cross-lagged paths reached statistical significance (see Table 1). Notably, among the 16 cross-lagged paths examined across all four frailty dimensions and two time intervals, only two paths survived FDR correction: one from psychological frailty T1 to weekly mobile phone call time T2 (*P*_*FDR* = 0.048) and one from social frailty T2 to weekly mobile phone call time T3 (*P*_*FDR* = 0.033). Neither path was replicated across intervals, and both effect sizes were small (β = 0.086 and β = 0.078, respectively), indicating that these associations are weak.

For psychological frailty, the cross-lagged paths did not support a robust bidirectional temporal pattern after FDR correction. Although weekly mobile phone call time at T2 showed a positive association with psychological frailty at T3 before correction (β = 0.067, *SE* = 0.027, *P* = 0.010), this effect did not survive FDR correction (*P_FDR* = 0.069). Similarly, the path from psychological frailty T2 to weekly mobile phone call time T3 was not significant after correction (β = 0.053, *SE* = 0.023, *P* = 0.025, *P_FDR* = 0.101). Thus, a mutually reinforcing cycle between psychological frailty and weekly mobile phone call time was not supported in this study.

Physical frailty showed no significant cross-lagged associations with weekly mobile phone call time at either interval (T1 → T2: β = 0.035, *SE* = 0.048, *P* = 0.465; T2 → T3: β = 0.052, *SE* = 0.029, *P* = 0.075; both *P_FDR* > 0.05). The previously reported positive association from physical frailty T2 to weekly mobile phone call time T3 was not statistically significant in the present data after correction. Social frailty at T2 was positively associated with subsequent weekly mobile phone call time at T3 (β = 0.078, *SE* = 0.025, *P* = 0.002, *P_FDR* = 0.033), indicating a temporal direction from social frailty to increased phone use during this interval only. No significant effect was found for the opposite direction (mobile phone T2 → social frailty T3: β = 0.021, *SE* = 0.019, *P* = 0.285). No temporal relationship was found between cognitive frailty and weekly mobile phone call time in either direction, as none of the cross-lagged paths reached statistical significance (all *P* > 0.05), indicating that cognitive frailty and weekly mobile phone call time are not temporally related in either direction. Given the reverse scoring of cognitive frailty (higher score = better cognition), these null findings are not attributable to directionality artifacts.

Overall, the cross-lagged results reveal a sparse pattern: only two of 16 tested paths were significant after FDR correction, both were observed in only one time interval, and neither showed evidence of replication across intervals. These findings do not support consistent temporal associations between weekly mobile phone call time and the four frailty dimensions.

#### 3.2.3 Random intercepts and covariances

As shown in Table 1, the random intercept variance for weekly mobile phone call time was significant in all four models (all *SE* ≤ 0.021, all *P* < 0.001), indicating substantial stable between-person differences in phone use over time. This does not imply the absence of within-person variation; rather, it reflects the proportion of total variance attributable to time-invariant inter-individual heterogeneity.

Regarding the random intercept variances for frailty dimensions, the random intercepts for physical frailty (β = 0.449, *SE* = 0.032, *P* < 0.001), psychological frailty (β = 0.571, *SE* = 0.031, *P* < 0.001), cognitive frailty (β = 0.462, *SE* = 0.032, *P* < 0.001), and social frailty (β = 0.388, *SE* = 0.019, *P* < 0.001), indicating that all four frailty dimensions exhibited substantial trait-like stability at the between-person level.

The covariances between the random intercepts of each frailty dimension and weekly mobile phone call time were uniformly non-significant (all *P* > 0.05).

Regarding residual covariances at the within-person level (i.e., concurrent associations between frailty and mobile phone at each time point), the following patterns were observed: for physical frailty, none reached significance (T1: *P* = 0.748; T2: *P* = 0.060; T3: *P* = 0.174); for psychological frailty, significant associations were found at T1 (β = 0.073, *SE* = 0.025, *P* = 0.004), T2 (β = 0.097, *SE* = 0.030, *P* = 0.001), and T3 (β = 0.048, *SE* = 0.021, *P* = 0.021); for cognitive frailty, none reached significance (T1: *P* = 0.404; T2: *P* = 0.720; T3: *P* = 0.799); for social frailty, a significant association was found only at T3 (β = 0.036, *SE* = 0.018, *P* = 0.046), but not at T1 (*P* = 0.893) or T2 (*P* = 0.394).Taken together, these findings suggest that the temporal associations between weekly mobile phone call time and frailty operate primarily at the within-person level, with significant concurrent couplings observed most consistently for psychological frailty across all three time points, and for social frailty at T3. However, the significant random intercepts for both weekly mobile phone call time and all frailty dimensions also indicate substantial stable inter-individual differences that should not be overlooked.

## 4 Discussion

At the uncorrected *P*-value level, psychological frailty showed a trend toward bidirectional associations with weekly mobile phone call time across multiple intervals (XF T1 → WCT T2: β = 0.086, *SE* = 0.031, *P* = 0.008; WCT T2 → XF T3: β = 0.067, *SE* = 0.027, *P* = 0.010; XF T2 → WCT T3: β = 0.053, *SE* = 0.023, *P* = 0.025). However, after FDR correction, only the path from psychological frailty T1 to weekly mobile phone call time T2 remained statistically significant (β = 0.086, *SE* = 0.031, *P_FDR* = 0.048), whereas the adjusted *P*-values for the other two paths exceeded 0.05 (*P_FDR* = 0.069 and 0.101, respectively). In the reverse direction, the predictive effect of weekly mobile phone call time on psychological frailty was not robustly supported after correction; no clear longitudinal associations were observed between physical or cognitive frailty and weekly mobile phone call time (all *P_FDR* > 0.05). The path from social frailty T2 to weekly mobile phone call time T3 was another significant pathway after correction (β = 0.078, *SE* = 0.025, *P_FDR* = 0.033), suggesting that individuals with insufficient social participation may compensate for social deficits by increasing mobile phone use. Collectively, these findings indicate that the temporal associations between weekly mobile phone call time and multidimensional frailty in middle-aged and older adults are clearly dimension-specific; however, after adjustment for multiple comparisons, only the predictive effects of psychological frailty (T1 → T2) and social frailty (T2 → T3) on subsequent weekly mobile phone call time received some support, while evidence for bidirectional associations remained insufficient. Crucially, both significant paths appeared in different time intervals, and neither was replicated across intervals, indicating that these associations may be interval-specific rather than stable temporal patterns. Moreover, both effect sizes were small (β = 0.086 and β = 0.078), and the overall pattern across 16 cross-lagged tests was sparse, with only two paths surviving FDR correction. Therefore, these findings should not be interpreted as evidence of robust or consistent temporal relationships.

The temporal association pattern between psychological frailty and weekly mobile phone call time, after FDR correction, exhibited a possible early-stage unidirectional driving feature: early psychological frailty (T1) was robustly associated with subsequent increases in weekly mobile phone call time, whereas the reverse association from call time to later psychological frailty (T3) was only marginally significant, and the driving effect from later psychological frailty to call time was not significant. This is partially consistent with the findings of Karaş et al. (20) and Wei et al. (21). One speculative explanation is that when psychological frailty worsens in middle-aged and older adults, it is often accompanied by depressive symptoms, elevated anxiety levels, and impaired sleep quality. The potential mechanisms can be considered from the perspective of geropsychology. When psychological frailty worsens in middle-aged and older adults, it is often accompanied by prominent depressive symptoms, elevated anxiety levels, and persistently impaired sleep quality. During the aging process, with shrinking social circles and limited channels for emotional regulation in real life, they may turn to mobile phones—a low-cost and easily accessible tool (22)—to maintain online social connections, divert negative feelings, seek emotional comfort, and alleviate loneliness, which may lead to increased frequency and duration of weekly mobile phone call time (23). Conversely, the reverse association from increased call time to psychological frailty was only weakly manifested at the later stage and did not survive strict correction, possibly because the cumulative effect of calling behavior may require a longer period to produce a detectable temporal association with worsening psychological frailty, or it may be limited by the sensitivity of call duration as a single indicator. However, it is important to emphasize that these mechanisms were not directly measured in the present study. Factors such as depressive symptoms, anxiety, sleep quality, emotional regulation, and loneliness were not specifically examined as mediators, and the term “psychological frailty” itself encompasses some of these constructs. Therefore, the above interpretation remains speculative and should be tested in future studies with appropriate mediation designs and more refined measures of psychological states. For example, prolonged weekly mobile phone use may encroach on time for offline socializing, outdoor activities, and daily functional activities in this population, thereby potentially exacerbating real-life social isolation. Similarly, constructs such as online information overload, social comparison, short-video use, and reduced offline activity—while plausible—were not measured in our study and should be considered as hypotheses for future research rather than explanations for our findings (24). These pathways may collectively delineate a temporal pattern suggestive of early psychological frailty—that is, the emergence of psychological frailty may promote increased call time, but given that the reverse association was not supported and the significant path was not replicated, it is difficult to confirm a unidirectional, let alone bidirectional, cycle.

Unlike the psychological dimension, social frailty (in the later stage) showed a unidirectional temporal association with subsequent weekly mobile phone call time. Under strict FDR correction, later-stage social frailty (T2 → T3) was robustly positively associated with subsequent call time (β = 0.078, *SE* = 0.025, *P* = 0.002, *P_FDR* = 0.033). One interpretation, consistent with the social compensation hypothesis, is that middle-aged and older adults with insufficient social participation and shrunken social networks tend to use mobile phones more frequently, which may explain the positive temporal association between social frailty and subsequent weekly mobile phone call time. However, as this association was observed only in the later interval (T2 → T3) and not replicated in the earlier interval (T1 → T2), this interpretation should be treated with caution. The absence of a reverse temporal association from call time to social frailty (*P*_*FDR* > 0.05) may stem mainly from two reasons. On the one hand, for middle-aged and older adults in the later stage of social frailty, mobile calls are mostly for maintaining existing connections; such lightweight virtual contact may not readily translate into high-quality offline social participation or provide substantial social support, and thus may fail to fundamentally expand their social networks or repair their declining social functions (25). On the other hand, social frailty exhibited extremely high autoregressive stability from T2 to T3 (β = 0.438, *P* < 0.001), suggesting that its developmental trajectory may have become relatively stable. At this stage, the decline in social functioning appears to have entered a relatively fixed path, and daily call duration alone, as a single behavioral variable, may be insufficient to effectively alter this process. Nevertheless, these explanations remain speculative, and the single-interval nature of the finding limits the strength of any causal or temporal inference.

However, the findings of this study seem to differ from those of Keränen et al. (26) on information and communications technology (ICT) use among frail community-dwelling older adults in Finland. That study adopted a cross-sectional design and only analyzed the association between physical frailty and advanced mobile ICT use at a single time point, reporting a negative correlation between frailty status and digital technology use level, and that frail older adults held more negative attitudes toward the practicality and usability of mobile ICT. In contrast, the present study employed a cohort design and examined the dynamic temporal relationship between physical frailty and weekly mobile phone call time through longitudinal follow-up, which may effectively overcome the limitations of cross-sectional studies that cannot distinguish temporal order and are susceptible to confounding. The differences in findings may relate to several factors: first, regional differences in the sociocultural background and digital inclusion support systems of the study populations; second, the present study focused on the dependency on and compensatory needs for weekly mobile phone call time among middle-aged and older adults, while the aforementioned study paid more attention to the overall penetration rate and active adoption willingness of ICT use. In addition, differences in frailty assessment tools and measurement dimensions of digital use behavior collectively suggest that the association between physical frailty and digital technology use in this population may not be linear but could be jointly influenced by multiple factors such as study design, cultural environment, usage scenarios, and social support.

No significant bidirectional temporal association was identified between cognitive frailty and weekly mobile phone call time in this study. However, the absence of a significant association does not demonstrate that weekly mobile phone call time and cognitive frailty evolved independently or were not temporally related. Rather, it indicates that, within the limitations of our measurement and study design, we did not find evidence for such an association. Possible explanations are as follows. First, the core features of cognitive frailty involve declines in executive function and memory, which may limit the ability to operate smartphones in complex ways. Thus, even when there is an emotional need, effective interaction via mobile phones may be difficult, reducing the feasibility of using call time as a coping tool. Meanwhile, individuals with lower cognitive levels tend to maintain simple and fixed usage habits (e.g., only answering calls) rather than actively increasing frequency or duration. Second, from the reverse pathway, the potential impact of call time on cognitive function may be subject to a “threshold effect” or “content specificity”—if calls involve only routine simple communication rather than cognitively demanding interactions, their association with cognitive function may be very limited. However, because the present study measured only call duration without information on the nature of calls, and because the model fit for the cognitive frailty model was suboptimal (TLI = 0.860). These remain speculations that warrant further investigation with more refined digital behavior indicators.

From a methodological perspective, the age range of the sample in this study was relatively broad (40–70 years), and some participants had not yet entered the advanced old-age stage characterized by rapid cognitive decline, which may have resulted in relatively limited incidence and variability of frailty. This “restricted range” effect may make it difficult for the model to capture the potential constraining effect of frailty on call time. Although the cognitive frailty dimension showed sufficient inter-individual heterogeneity in the sample (significant random intercept), its cross-lagged paths with call time remained non-significant, suggesting that we did not find evidence of the two evolve independently over time rather than demonstrating that they evolve independently. Thus, the potential dynamic associations in the oldest-old population might be underestimated or even masked. Therefore, future research needs to be further validated in the oldest-old population.

The present study has several limitations in the measurement of weekly mobile phone call time. The core exposure variable was constructed solely from self-reported call duration, failing to cover core dimensions of modern mobile phone use such as smartphone application usage and social media interaction. Considering that the data came from the UK Biobank (2006–2019) with an early collection time, it may objectively not reflect the full picture of contemporary smartphone use. Therefore, the conclusions of this study should be strictly limited to “weekly mobile phone call time” and cannot be generalized to “mobile phone use” or “digital behavior” in a broader sense. Furthermore, the exposure variable was derived from a single self-reported item with six ordered categories (ranging from “Less than 5 mins” to “More than 6 h”). These categories were assigned numerical scores from 1 to 6 and treated as a continuous variable after standardization to baseline. However, the intervals between these categories are not equally spaced (e.g., the difference between “Less than 5 mins” and “5–29 mins” is not equivalent to that between “1–3 h” and “4–6 h”), and standardization does not resolve this underlying inequality. While this coding approach has been used in previous UK Biobank studies, we acknowledge that treating ordinal categorical data as continuous may introduce measurement imprecision. Future research should employ sensitivity analyses using ordinal models or alternative duration-based codings (e.g., mid-point values or log-transformed hours) to examine whether the observed temporal associations are robust to different specifications of call duration. Future research is necessary to use refined measurement tools covering multidimensional usage behaviors and further examine the temporal associations between frailty and digital technology use in age-specific samples with more contemporary characteristics.

Overall, this study constructed a random intercept cross-lagged panel model and examined the temporal associations between weekly mobile phone call time and various frailty dimensions at the within-person level based on three-wave follow-up data, providing unique empirical insights into the longitudinal relationship between calling behavior and multidimensional frailty in middle-aged and older adults. Under strict FDR correction, the results showed that early psychological frailty (T1 → T2) and later-stage social frailty (T2 → T3) each showed a single-interval association with subsequent call time; however, neither path was replicated across intervals, and effect sizes were small. All paths for physical and cognitive frailty did not survive correction, and the overall pattern was sparse. Given that only two of 16 cross-lagged paths were significant—each in only one interval and with small effect sizes—these findings do not support consistent or robust temporal relationships between weekly mobile phone call time and most frailty dimensions. This dimension-specific association pattern is broadly consistent with the symptom network structure of multidimensional frailty (27). The present study, through its longitudinal design, overcomes the temporal limitations of previous cross-sectional network analyses, suggesting as a hypothesis that focusing on changes in early psychological frailty combined with enhanced social support may have greater potential value than merely adjusting call duration. It should be noted that these temporal associations should not be interpreted as causal and may be subject to potential confounding by structural factors such as digital exclusion and socioeconomic inequality. Furthermore, the non-replication of the two significant paths across intervals, the suboptimal model fit for several dimensions, the residual confounding from unmeasured variables, the uncertainty inherent in the operationalisation of the four frailty constructs (which were derived from different conceptual frameworks), and the restricted measurement of phone use (limited to self-reported call duration) collectively warrant caution in interpreting the present findings. Compared with participants excluded due to missing data, the analytical sample showed significant differences in age, sex, ethnicity, and retirement status, while no marked differences were observed in other baseline characteristics, indicating the presence of healthy volunteer bias in this study. The included participants were likely to be healthier, which may affect the generalizability of the findings to the general middle-aged and older population to some extent.

Furthermore, sample selection and follow-up attrition may introduce bias. Due to the small sample size of participants aged 65 and older in this study, age subgroup analyses could not be conducted; the majority of the sample was under 65 years old, and this age composition may limit the generalizability of the conclusions to older age groups. The analytical sample included only 5,533 participants with complete data across all three assessments and no missing values for call time or frailty dimensions. Compared with participants excluded due to incomplete data, our analytical sample was younger, had more males, and lower unemployment/retirement rates, suggesting that this highly selected sample may be healthier, more willing to participate, and have better digital access, which may introduce healthy volunteer bias, survivor bias, and digital access bias. Participants who remain in a longitudinal study over multiple waves tend to be healthier and more socially engaged, which may attenuate the observed associations between frailty and phone use. Conversely, those who are frail, have lower socioeconomic status, or have limited digital literacy may be less likely to own mobile phones or to continue participating, potentially leading to an underestimation of the true associations in the general population. In particular, digital access bias may selectively exclude individuals who are less familiar with or have less access to mobile technology, thereby restricting the variability of phone use in our sample and limiting the generalisability of our findings to populations with limited digital engagement. Although the present study used full information maximum likelihood estimation to handle missingness in primary variables, sensitivity analyses such as inverse probability weighting or pattern-mixture models were not conducted for the selective inclusion due to complete-case exclusion, which may limit the generalizability of the results to the general middle-aged and older population. Future research should test the robustness of these temporal associations under more flexible missing-data handling strategies (e.g., multiple imputation with auxiliary variables) and provide detailed comparisons of baseline characteristics between included and excluded participants to evaluate the possible impact of selection bias.

## Data Availability

The original contributions presented in the study are included in the article/Supplementary material, further inquiries can be directed to the corresponding authors.
