# Supplementary material for: Relationship between mobile phone use and multidimensional frailty: evidence from UK Biobank
Source: Front Public Health. 2026 Aug 10;14:1868178. doi: 10.3389/fpubh.2026.1868178 (PMC13500240; doi:10.3389/fpubh.2026.1868178)
Supplement: Supplementary file 1 [file Supplementary_file_1.docx]

**Supplementary Table 1**

*Participant Demographic Information at Each Wave*

| **Demographic Characteristic** | *Baseline*  *n* (%) | *Time 1*  *n* (%) | *Time 2*  *n* (%) | |  |
| --- | --- | --- | --- | --- | --- |
| Sex |  |  |  | |  |
| *Male* | 2701 (48.82) | - | - | |  |
| *Female* | 2832 (51.18) | - | - | |  |
| Ethnicity |  |  |  | |  |
| *White* | 5379 (97.22) | - | - | |  |
| *Asian* | 64 (1.16) | - | - | |  |
| *Black* | 36 (0.65) | - | - | |  |
| *Mixed* | 20 (0.36) | - | - | |  |
| *Don’t know/Prefer not to say* | 10 (0.18) | - | - | |  |
| *Other* | 24 (0.43) | - | - | |  |
| Townsend Deprivation Index (Baseline) Mean ± SD | -1.92 ± 2.66 |  |  | |  |
| Has disease (Baseline only) |  |  |  | |  |
| *Has disease* | 3166 (57.22) |  |  | |  |
| *No disease* | 2367 (42.78) |  |  | |  |
| Employment/Retirement |  |  |  | |  |
| *Employed* |  |  |  | |  |
| *Retired/Unemployed* | 1136 (20.6) |  |  | |  |
| *Missing* | 29 (0.5) |  |  | |  |
| Baseline psychological frailty (XF0) |  |  |  | |  |
| *Mean* ± *SD* | 0.21 ± 0.20 |  |  | |  |
| *Median [IQR]* | 0.14 [0.00, 0.33] |  |  | |  |
| Alcohol Consumption |  |  |  | |  |
| *Daily/Almost Daily* | 1138 (20.57) | 842 (15.22) | 822 (14.86) | |  |
| *Other* | 4395 (79.43) | 4691 (84.78) | 4711 (85.14) | |  |
| Smoking |  |  |  | |  |
| *Current smoker* | 320 (5.78) | 175 (3.16) | 128 (2.31) | |  |
| *Other* | 5213 (94.22) | 5358 (96.84) | 5405 (97.69) | |  |
| BMI (kg/m²) Mean ± SD | 26.59 ± 4.10 | 26.61 ± 4.28 | 26.65 ± 4.38 | |  |
| Living alone |  |  |  | |  |
| *Yes* | 777 (14.0) | 984 (17.8) | 1068 (19.3) | |  |
| *No* | 4756 (86.0) | 4542 (82.1) | 4455 (80.5) | |  |
| *Missing* | 0 | 7 (0.1) | 10 (0.2) | |  |
| Social network score (0-1) |  |  |  | |  |
| *Mean* ± *SD* | 0.35 ± 0.21 | 0.33 ± 0.21 | 0.34 ± 0.23 | |  |
| *Median [IQR]* | 0.40 [0.20, 0.40] | 0.40 [0.20, 0.40] | | 0.40 [0.20, 0.40] | |
| *Missing* | 12 (0.2) | 5 (0.1) | 28 (0.5) | |  |

*Note. All values are based on the full sample (N=5533) at each wave where data were available. Dashes (–) indicate not assessed or not applicable at that wave. “Other” in ethnicity includes any response not classifiable into the listed categories. T2 corresponds to Instance 2, T3 corresponds to Instance 3*

**Supplementary Table 2 - Frailty Categorisation and Coding**

*All items within each frailty dimension were equally weighted. The composite score for each dimension was calculated as the mean of non-missing items (mean-score method). For each dimension, a composite score was computed only if the participant completed at least the following number of items: physical frailty: 9 of 11 items; psychological frailty: 5 of 7 items; cognitive frailty: 3 of 4 items; social frailty: 5 of 7 items. For physical, psychological, and social frailty, higher scores indicate greater frailty. For cognitive frailty, higher scores indicate better cognitive function (i.e., lower frailty).*

*Physical Frailty*

Low handgrip strength, exhaustion, low physical activity, perceived health, walking, and poor hearing and vision

| **Subdomain** | **Question** | **Category/Field** | **Coding** |
| --- | --- | --- | --- |
| Low Handgrip Strength | Handgrip – left/right used depending on handedness | Handgrip 100019/46 &47  Handedness  100033/1707  BMI 100010/21001 | Cut-off points for handgrip strength in kg stratified by sex and BMI based on the Fried Phenotype (Fried et al., 2001).  Males received a one if hand grip strength ≤29.0 kg for BMI ≤24.0, ≤30.0 kg for BMI 24.1-26.0, ≤30 kg for BMI 26.1-28.0, and ≤32.0 kg for BMI >28.0.  Females received a one if hand grip strength ≤17.0 kg for BMI ≤23.0, ≤17.3 kg for BMI 23.1-26.0, ≤18.0 kg for BMI 26.1-29.0, and ≤21.0 kg for BMI >29.0 |
| Exhaustion | “Over the past two weeks, how often have you felt tired or had little energy?” | 100060/2080 | Not at all (0), several days (0.33), more than half (0.67), nearly every day (1) |
| Low Physical Activity | International Physical Activity Questionnaire (IPAQ Research Committee, 2005) low activity group assignment | 100054/22032 | Low (1), moderate (0), high (0) |
| Perceived Health | “In general how would you rate your overall health?” | 100042/2178 | Excellent (0), good (0. 33) fair (0.67) and poor (1) |
| Walking | 1. “Do you get short of breath walking with people of your own age on level ground?”  2. “Do you get a pain in either leg on walking?”  3. “How would you describe your usual walking pace?” | 1  100037/4717  2  100038/4728  3  100054/924 | 1 & 2: Yes (1), No (0)  3: Not collected for people who indicated that they are unable to walk, thus NA (1), slow pace (1), steady average pace (0), brisk pace (0), None of the above (1) |
| Poor Hearing | 1. “Do you have any difficulty with your hearing?”  2. “Do you find it difficult to follow a conversation if there is background noise (such as TV, radio, children playing)” | 100043/2247 & 2257 | 1: I am completely deaf (1), Yes (1), No (0)  2: If listed “I am completely deaf” in above (1), otherwise Yes (1), No (0) |
| Poor Vision | 1. “Do you wear glasses or contact lenses to correct your vision?”  2. “Has a doctor told you that you have any of the following problems with your eyes? [diabetes related eye disease, glaucoma, injury/trauma resulting in loss of vision, cataract, macular degeneration, other]” | 100041/2207 & 6148 | 1: Yes (1), No (0)  2: Allow multiple answers, however we only coded the first mention of disease (as interested in if have, not how many or what). Any disease, including “other” (1), None of the above (0) |

*Note.* Prefer not to answer and do not know recoded to 0 (i.e., no frailty).

*Psychological Frailty*

Depressive symptoms, anxiety symptoms and coping with problems

| **Subdomain** | **Question** | **Category/Field** | **Coding** |
| --- | --- | --- | --- |
| Depressive Symptoms | 1. “Do you ever feel 'just miserable' for no reason?”  2. “Over the past two weeks, how often have you felt down, depressed or hopeless?”  3. “Over the past two weeks, how often have you had little interest or pleasure in doing things?” | 100060/1930, 2050 & 2060 | 1: Yes (1), No (0)  2 & 3: Not at all (0), several days (0.33), more than half (0.67), nearly every day (1) |
| Anxiety Symptoms | 1. “Do you suffer from 'nerves'?”  2. “Over the past two weeks, how often have you felt tense, fidgety or restless?” | 100060/2010 & 2070 | 1: Yes (1), No (0)  2: Not at all (0), several days (0.33), more than half (0.67), nearly every day (1) |
| Coping with Problems | 1. “Are you a worrier?”  2. “Would you call yourself tense or 'highly strung'” | 100060/1980 & 1990 | 1 & 2: Yes (1), No (0) |

*Note.* Prefer not to answer and do not know recoded to 0 (i.e., no frailty).

*Cognitive Frailty*

Measures of reaction time, fluid intelligence/reasoning, and visual and numeric memory

| **Subdomain** | **Question** | **Category/Field** | **Coding** |
| --- | --- | --- | --- |
| Reaction Time | Timed test of symbol matching | 100032/20023 | Mean response time in milliseconds across trials which contained matching pairs |
| Fluid Intelligence/Reasoning | Thirteen verbal and numerical reasoning questions (e.g., addition, word interpolation, square sequence recognition) and included a two-minute limit | 100027/20016 | Fluid intelligence score (0-13) |
| Visual Memory | A pairs matching task (participants memorizing the position of six card pairs and then being asked to match them from memory with as few errors as possible) | 100030/399 | Number of incorrect matches^a^ |
| Numeric Memory | Numerical string recall (become sequentially longer) | 100029/4282 | Maximum digits correctly remembered |

*Note.* Prefer not to answer and do not know recoded to 0 (i.e., no frailty). Due to the longitudinal nature of the present study, cognitive assessments were only considered for inclusion if they had been assessed on three instances (0, 2, 3). Additionally, the prospective memory task was omitted due to the dichotomous nature of the task (i.e., did not capture differing degrees of prospective memory functioning).

^a^ Deviation from pre-registration where stated number of correct matches. Incorrect matches more accurate gauge of memory. This score was inverse scored to be in the same direction as other measures.

*Social Frailty*

Living alone, social network, social support, loneliness, and social activities

| **Subdomain** | **Question** | **Category/Field** | **Coding** |
| --- | --- | --- | --- |
| Living Alone | “Including yourself, how many people are living together in your household?” | 100066/709 | 1 (1), any other number (0) |
| Social Network | “How often do you visit friends or family or have them visit you?” | 100061/1031 | Almost daily (0), 2-4 times a week (0.20), about once a week (0.40), about once a month (0.60), once every few months (0.80), never or almost never (1). If participants selected “no friends/family outside the household” and reported living alone (1). otherwise (.20) |
| Social Support | 1. “How often are you able to confide in someone close to you?”  2. “In general how satisfied are you with your family relationships”  3. “In general how satisfied are you with your friendships” | 1  100061/2110  2 & 3  100060/4559 & 4570 | 1: Almost daily (0), 2-4 times a week (0.20), about once a week (0.40), about once a month (0.60), once every few months (0.80), never or almost never (1)  2 & 3: Extremely happy (0), very happy (0.20), moderately happy (0.40), moderately unhappy (0.60), very unhappy (0.80), extremely unhappy (1) |
| Loneliness | “Do you often feel lonely?” | 100060/2020 | Yes (1), No (0) |
| Social Activities | “Which of the following do you attend once a week or more often? [sports club/gym, pub/social club, religious group, adult education class, other]” | 100061/6160 | None of the social activities listed (1), one social activity (0.80), two social activities (0.60), three social activities (0.40), four social activities (0.20), five social activities (0)^a^ |

*Note.* Prefer not to answer and do not know recoded to 0 (i.e., no frailty).

^a^ This deviates from pre-registration which was incorrectly coded (i.e., higher score did not reflect greater social frailty

**Supplementary Table 3**

*Weekly mobile phone call time Score Categorisation and Standardisation*

| **Subdomain** | **Question** | **Category/Field** | **Coding** |
| --- | --- | --- | --- |
| Weekly usage of mobile phone in last 3 months | “Over the last 3 months, on average how much time per week did you spend making or receiving calls on a mobile phone?” | 100053/1120 | Less than 5 mins (1), 5–29 mins (2), 30–59 mins (3), 1–3 hours (4), 4–6 hours (5), More than 6 hours (6). Do not know or prefer not to answer were coded as NA. |

**Supplementary Table 4**

*Bivariate Correlations between demographic variables, frailty dimensions, and weekly mobile phone call time.*

|  | | **Frailty Dimensions** | | | | | | | | | | | | | | **Weekly mobile phone call time** | | |
| --- | --- | --- | --- | --- | --- | --- | --- | --- | --- | --- | --- | --- | --- | --- | --- | --- | --- | --- |
|  |  | 1 | 2 | 3 | 4 | 5 | 6 | 7 | 8 | 9 | 10 | 11 | 12 |  | 1 | | 2 | 3 |
| **Demographics** | 1. Age | 0.22*** | 0.21*** | 0.22*** | -0.11*** | -0.16*** | -0.14*** | 0.18*** | 0.22*** | 0.27*** | -0.07** | -0.05*** | -0.02 |  | -0.20*** | | -0.21*** | -0.17*** |
|  | 2. Male | 0.01 | 0.06*** | 0.05** | -0.10*** | -0.12*** | -0.14*** | -0.08* | -0.07*** | -0.06*** | 0.08** | 0.03* | 0.01 |  | 0.09*** | | -0.05*** | -0.10*** |
|  | 3. Tdi | 0.07* | 0.06*** | 0.06*** | 0.07*** | 0.07*** | 0.07*** | -0.02 | 0.05*** | 0.02 | 0.15*** | 0.14*** | 0.14*** |  | 0.04** | | 0.06*** | 0.05*** |
|  | 4. Disease | 0.20*** | 0.19*** | 0.20*** | 0.05*** | 0.04** | 0.04** | 0.02 | 0.04* | 0.07*** | 0.03 | 0.02 | 0.03* |  | -0.01 | | -0.03* | -0.04** |
|  | 5. British | -0.02 | 0.01 | 0.00 | 0.00 | 0.00 | 0.01 | -0.06 | -0.05*** | -0.06*** | -0.06** | -0.05** | -0.04** |  | -0.03* | | -0.05*** | -0.07*** |
|  | 6. Retired | 0.01 | 0.00 | 0.01 | 0.00 | 0.00 | 0.00 | 0.01 | 0.01 | 0.01 | -0.05* | -0.05* | -0.04* |  | -0.07* | | 0.00 | 0.00 |
|  | 7. Living alone | 0.00 | 0.00 | 0.00 | 0.03 | 0.03 | 0.03 | 0.00 | 0.01 | 0.00 | 0.15* | 0.14* | 0.14* |  | 0.01 | | 0.00 | 0.00 |
|  | 8. Social network | -0.05* | -0.08* | -0.09* | 0.03 | 0.04 | 0.04 | -0.01 | -0.01 | -0.01 | 0.02 | 0.03 | 0.03 |  | 0.03 | | 0.04 | 0.05 |
|  | 9. Smoke.1 | 0.00 | 0.03* | 0.03* | 0.05*** | 0.03* | 0.02 | 0.03 | 0.01 | 0.03* | 0.05* | 0.07*** | 0.05*** |  | 0.06*** | | 0.04** | 0.02 |
|  | 10. Smoke.2 | 0.04 | 0.02 | 0.03* | 0.03* | 0.02 | 0.02 | -0.02 | 0.01 | 0.00 | 0.02 | 0.05*** | 0.04** |  | 0.04** | | 0.03* | 0.01 |
|  | 11. Smoke.3 | -0.03 | 0.02 | 0.02 | 0.00 | 0.01 | 0.01 | -0.03 | -0.01 | -0.01 | 0.01 | 0.05*** | 0.02 |  | 0.03 | | 0.02 | 0.02 |
|  | 12. Alcohol.1 | -0.03 | 0.00 | 0.02 | -0.03* | -0.03* | -0.02 | -0.02 | -0.04** | -0.02 | 0.00 | 0.00 | 0.01 |  | -0.01 | | -0.03* | -0.05*** |
|  | 13. Alcohol.2 | 0.03 | 0.01 | 0.02 | -0.04** | -0.03* | -0.03* | 0.01 | -0.03* | -0.02 | -0.02 | 0.00 | -0.01 |  | 0.00 | | -0.02 | -0.04** |
|  | 14. Alcohol.3 | 0.03 | 0.01 | 0.02 | -0.05*** | -0.04** | -0.03* | -0.02 | -0.04** | -0.02 | -0.02 | -0.01 | -0.02 |  | -0.02 | | -0.03* | -0.04** |
|  | 15. BMI.1 | 0.30*** | 0.25*** | 0.24*** | -0.01 | -0.03* | -0.03* | -0.02 | 0.02 | 0.02 | 0.09*** | 0.05*** | 0.07*** |  | 0.07*** | | 0.03 | 0.00 |
|  | 16. BMI.2 | 0.26*** | 0.28*** | 0.27*** | 0.01 | 0.00 | -0.01 | 0.01 | 0.03* | 0.02 | 0.11*** | 0.06*** | 0.08*** |  | 0.09*** | | 0.07*** | 0.03* |
|  | 17. BMI.3 | 0.25*** | 0.26*** | 0.27*** | 0.03 | 0.01 | 0.01 | -0.01 | 0.03* | 0.01 | 0.12*** | 0.08*** | 0.09*** |  | 0.10*** | | 0.08*** | 0.04** |
| **Frailty Dimensions** | 1. Physical.1 |  | 0.62*** | 0.58*** | 0.21*** | 0.15*** | 0.17*** | 0.02 | 0.05 | 0.10** | 0.14*** | 0.08* | 0.05 |  | -0.07* | | -0.01 | -0.02 |
|  | 2. Physical.2 |  |  | 0.72*** | 0.21*** | 0.23*** | 0.21*** | 0.09* | 0.10*** | 0.11*** | 0.15*** | 0.17*** | 0.18*** |  | -0.02 | | -0.01 | 0.00 |
|  | 3. Physical.3 |  |  |  | 0.20*** | 0.21*** | 0.24*** | 0.10* | 0.09*** | 0.10*** | 0.13*** | 0.17*** | 0.20*** |  | -0.05* | | -0.02 | -0.01 |
|  | 4. Psychological.1 |  |  |  |  | 0.68*** | 0.65*** | 0.09** | 0.07*** | 0.07*** | 0.29*** | 0.25*** | 0.23*** |  | 0.03 | | 0.05*** | 0.03* |
|  | 5. Psychological.2 |  |  |  |  |  | 0.76*** | 0.08* | 0.05** | 0.05** | 0.20*** | 0.31*** | 0.26*** |  | 0.01 | | 0.05*** | 0.05*** |
|  | 6. Psychological.3 |  |  |  |  |  |  | 0.10** | 0.04** | 0.05** | 0.23*** | 0.26*** | 0.31*** |  | -0.01 | | 0.05*** | 0.05*** |
|  | 7. Cognitive.1 |  |  |  |  |  |  |  | 0.60*** | 0.58*** | -0.05 | -0.05 | -0.03 |  | -0.01 | | 0.05 | 0.07* |
|  | 8. Cognitive.2 |  |  |  |  |  |  |  |  | 0.61*** | 0.02 | 0.01 | 0.02 |  | -0.04* | | -0.02 | 0.00 |
|  | 9. Cognitive.3 |  |  |  |  |  |  |  |  |  | 0.03 | 0.01 | 0.03 |  | -0.04* | | -0.01 | 0.00 |
|  | 10. Social.1 |  |  |  |  |  |  |  |  |  |  | 0.60*** | 0.58*** |  | 0.02 | | 0.04 | 0.07** |
|  | 11. Social.2 |  |  |  |  |  |  |  |  |  |  |  | 0.72*** |  | 0.03 | | 0.03* | 0.05*** |
|  | 12. Social.3 |  |  |  |  |  |  |  |  |  |  |  |  |  | 0.01 | | 0.03* | 0.05*** |
| **WCT** | 1. WCT.1 |  |  |  |  |  |  |  |  |  |  |  |  |  |  | | 0.46*** | 0.36*** |
|  | 2. WCT.2 |  |  |  |  |  |  |  |  |  |  |  |  |  |  | |  | 0.54*** |
|  | 3. WCT.3 |  |  |  |  |  |  |  |  |  |  |  |  |  |  | |  | - |

*Note.* **P* < 0.05, ** *P* < 0.01, *** *P* < 0.001. Non‑significant paths (*P* ≥ 0.05) are shown without asterisks. TDI = Townsend Deprivation Index; BMI = body mass index; PF = physical frailty; XF = psychological frailty; CF = cognitive frailty; SF = social frailty; WCT = weekly mobile phone call time. Frailty scores were calculated using the mean-score method (see Methods). For cognitive frailty (CF1–CF3), higher scores indicate better cognitive function. Social network is reverse-coded (higher scores indicate less frequent social contact). WCT scores were standardized to baseline (T1) using the baseline mean and standard deviation. Missing data were handled using pairwise deletion. Time points: 1 = T1 (baseline, 2006–2010), 2 = T2 (2014+), 3 = T3 (2019+). Retired (retired/unemployed), living alone, and social network were included as covariates in the RI-CLPM models (see Methods).

**Supplementary Table 5**

*Descriptive Statistics of Main Study Variables Across Three Waves (N = 5,533)*

|  | **N** | **Minimum** | **1^st^ Quartile** | **M (SD)** | **3^rd^ Quartile** | **Maximum** |
| --- | --- | --- | --- | --- | --- | --- |
| **Physical Frailty (0-11)** |  |  |  |  |  |  |
| Time 1 | 876 | 0.00 | 1.33 | 2.53 (1.42) | 3.33 | 8.67 |
| Time 2 | 4687 | 0.00 | 1.66 | 2.89 (1.51) | 3.66 | 10.00 |
| Time 3 | 4218 | 0.00 | 2.00 | 3.15 (1.61) | 4.33 | 10.00 |
|  |  |  |  |  |  |  |
| **Psychological Frailty (0-7)** |  |  |  |  |  |  |
| Time 1 | 5533 | 0.00 | 0.00 | 1.44 (1.39) | 2.33 | 7.00 |
| Time 2 | 5533 | 0.00 | 0.00 | 1.27 (1.37) | 2.00 | 7.00 |
| Time 3 | 5533 | 0.00 | 0.00 | 1.22 (1.35) | 2.00 | 7.00 |
|  |  |  |  |  |  |  |
| **Cognitive Frailty (z-scores)** |  |  |  |  |  |  |
| Time 1 | 900 | -1.70 | -0.38 | 0.01 (0.57) | 0.36 | 2.14 |
| Time 2 | 4687 | -2.07 | -0.41 | 0.00 (0.59) | 0.38 | 3.13 |
| Time 3 | 4006 | -2.00 | -0.43 | -0.03 (0.61) | 0.34 | 4.34 |
|  |  |  |  |  |  |  |
| **Social Frailty (0-7)** |  |  |  |  |  |  |
| Time 1 | 1739 | 0.60 | 1.40 | 2.06 (0.85) | 2.60 | 5.40 |
| Time 2 | 5264 | 0.20 | 1.40 | 2.09 (0.91) | 2.60 | 6.20 |
| Time 3 | 5210 | 0.20 | 1.40 | 2.13 (0.93) | 2.60 | 6.80 |
|  |  |  |  |  |  |  |
| **Weekly mobile phone call time (z-scores)** |  |  |  |  |  |  |
| Time 1 | 5508 | -1.25 | -0.50 | 0.00 (1.00) | 1.00 | 2.50 |
| Time 2 | 5500 | -1.25 | -0.50 | 0.03 (0.96) | 0.25 | 2.50 |
| Time 3 | 5495 | -1.25 | -0.50 | 0.11 (0.96) | 1.00 | 2.50 |

*Note.* Weekly mobile phone call time scores were standardised using baseline (Time 1) mean and standard deviation. Higher scores indicate more intensive use. For cognitive frailty, higher scores indicate better cognitive function (i.e., lower frailty). N represents the number of participants with non‑missing data at each time point.

**Supplementary Table 6**

*RI-CLPM Covariate Statistics*

|  | **Physical Frailty**  **and WCT** | | |  | **Psychological Frailty**  **and WCT** | | |  | **Cognitive Frailty**  **and WCT** | | |  | **Social Frailty**  **and WCT** | | |
| --- | --- | --- | --- | --- | --- | --- | --- | --- | --- | --- | --- | --- | --- | --- | --- |
|  | ***β*** | ***SE*** | ***P*** |  | ***β*** | ***SE*** | ***P*** |  | ***β*** | ***SE*** | ***P*** |  | ***β*** | ***SE*** | ***P*** |
| **Time 2 – Frailty** |  |  |  |  |  |  |  |  |  |  |  |  |  |  |  |
| Age | **0.237** | **0.037** | **<0.001** |  | 0.01 | 0.015 | 0.488 |  | **0.153** | **0.028** | **<0.001** |  | **0.172** | **0.021** | **<0.001** |
| Male Gender | 0.011 | 0.018 | 0.56 |  | **-0.15** | **0.021** | **<0.001** |  | **-0.104** | **0.025** | **<0.001** |  | 0.011 | 0.02 | 0.576 |
| TDI | **0.065** | **0.023** | **0.004** |  | **0.071** | **0.02** | **<0.001** |  | **0.094** | **0.02** | **<0.001** |  | **0.155** | **0.023** | **<0.001** |
| Disease | **0.149** | **0.029** | **<0.001** |  | **0.075** | **0.02** | **<0.001** |  | 0.009 | 0.02 | 0.634 |  | -0.026 | 0.019 | 0.168 |
| White British | 0.021 | 0.016 | 0.18 |  | **0.109** | **0.02** | **<0.001** |  | **-0.133** | **0.028** | **<0.001** |  | **0.043** | **0.018** | **0.017** |
| Retired | 0.019 | 0.019 | 0.335 |  | -0.010 | 0.021 | 0.636 |  | **0.089** | **0.023** | **<0.001** |  | **-0.086** | **0.019** | **<0.001** |
| Living alone | 0.011 | 0.019 | 0.571 |  | **0.076** | **0.015** | **<0.001** |  | **-0.084** | **0.023** | **<0.001** |  | — | — | — |
| Social network | **0.058** | **0.016** | **<0.001** |  | **0.085** | **0.016** | **<0.001** |  | **-0.057** | **0.018** | **0.002** |  | — | — | — |
| XF0 (baseline psych) | **0.263** | **0.036** | **<0.001** |  | — | — | — |  | **0.090** | **0.020** | **<0.001** |  | **0.310** | **0.027** | **<0.001** |
| T1 Daily Alcohol | -0.025 | 0.017 | 0.148 |  | 0 | 0.016 | 0.982 |  | **-0.045** | **0.019** | **0.016** |  | -0.014 | 0.017 | 0.411 |
| T1 Smoker | 0.021 | 0.017 | 0.209 |  | 0.015 | 0.016 | 0.364 |  | 0.014 | 0.02 | 0.483 |  | **0.054** | **0.017** | **0.002** |
| T1 BMI | — | — | — |  | **0.13** | **0.017** | **<0.001** |  | **-0.07** | **0.02** | **<0.001** |  | **0.209** | **0.027** | **<0.001** |
|  |  |  |  |  |  |  |  |  |  |  |  |  |  |  |  |
| **Time 2 – WCT** |  |  |  |  |  |  |  |  |  |  |  |  |  |  |  |
| Age | **-0.046** | **0.021** | **0.029** |  | **-0.101** | **0.013** | **<0.001** |  | **-0.102** | **0.016** | **<0.001** |  | **-0.098** | **0.015** | **<0.001** |
| Male Gender | **-0.052** | **0.016** | **0.001** |  | **-0.055** | **0.017** | **0.001** |  | **-0.065** | **0.018** | **<0.001** |  | **-0.07** | **0.017** | **<0.001** |
| TDI | 0.025 | 0.018 | 0.161 |  | 0.025 | 0.016 | 0.113 |  | **0.031** | **0.016** | **0.049** |  | 0.029 | 0.018 | 0.101 |
| Disease | -0.015 | 0.021 | 0.46 |  | -0.02 | 0.016 | 0.227 |  | -0.014 | 0.016 | 0.398 |  | -0.012 | 0.016 | 0.437 |
| White British | **0.039** | **0.015** | **0.009** |  | -0.02 | 0.015 | 0.194 |  | -0.005 | 0.018 | 0.798 |  | -0.011 | 0.015 | 0.459 |
| Retired (retired) | 0.019 | 0.019 | 0.335 |  | **-0.047** | **0.017** | **0.007** |  | **-0.043** | **0.018** | **0.014** |  | **-0.043** | **0.018** | **0.017** |
| Living alone | 0.011 | 0.019 | 0.571 |  | 0.025 | 0.015 | 0.091 |  | 0.024 | 0.015 | 0.099 |  | — | — | — |
| Social network | **0.058** | **0.016** | **<0.001** |  | **-0.056** | **0.016** | **<0.001** |  | **-0.057** | **0.016** | **<0.001** |  | — | — | — |
| XF0 (baseline psych) | **0.048** | **0.023** | **0.039** |  | — | — | — |  | **0.051** | **0.016** | **0.002** |  | 0.026 | 0.020 | 0.191 |
| T1 Daily Alcohol | -0.01 | 0.015 | 0.505 |  | -0.012 | 0.015 | 0.439 |  | -0.011 | 0.015 | 0.45 |  | -0.013 | 0.015 | 0.394 |
| T1 Smoker | 0.029 | 0.015 | 0.056 |  | 0.025 | 0.015 | 0.092 |  | 0.028 | 0.015 | 0.065 |  | 0.029 | 0.015 | 0.059 |
| T1 BMI | — | — | — |  | **0.13** | **0.014** | **<0.001** |  | **0.144** | **0.015** | **<0.001** |  | **0.137** | **0.02** | **<0.001** |
|  |  |  |  |  |  |  |  |  |  |  |  |  |  |  |  |
| **Time 3 – Frailty** |  |  |  |  |  |  |  |  |  |  |  |  |  |  |  |
| Age | **0.167** | **0.019** | **<0.001** |  | **0.032** | **0.013** | **0.015** |  | **0.155** | **0.023** | **<0.001** |  | **0.126** | **0.015** | **<0.001** |
| Male Gender | -0.002 | 0.014 | 0.868 |  | **-0.171** | **0.019** | **<0.001** |  | **-0.091** | **0.022** | **<0.001** |  | -0.008 | 0.015 | 0.61 |
| TDI | **0.052** | **0.015** | **<0.001** |  | **0.057** | **0.018** | **0.002** |  | **0.044** | **0.02** | **0.031** |  | **0.099** | **0.017** | **<0.001** |
| Disease | **0.1** | **0.018** | **<0.001** |  | **0.074** | **0.018** | **<0.001** |  | **0.06** | **0.019** | **0.002** |  | 0.003 | 0.015 | 0.849 |
| White British | -0.006 | 0.013 | 0.625 |  | **0.094** | **0.018** | **<0.001** |  | **-0.134** | **0.024** | **<0.001** |  | 0.023 | 0.014 | 0.109 |
| Retired (retired) | **0.034** | **0.014** | **0.017** |  | 0.032 | 0.020 | 0.104 |  | **0.095** | **0.020** | **<0.001** |  | -0.016 | 0.015 | 0.297 |
| Living alone | -0.011 | 0.013 | 0.379 |  | **0.058** | **0.015** | **<0.001** |  | **-0.097** | **0.016** | **<0.001** |  | — | — | — |
| Social network | **0.026** | **0.012** | **0.030** |  | **0.084** | **0.015** | **<0.001** |  | -0.007 | 0.015 | 0.645 |  | — | — | — |
| XF0 (baseline psych) | **0.149** | **0.018** | **<0.001** |  | — | — | — |  | **0.041** | **0.018** | **0.020** |  | **0.168** | **0.019** | **<0.001** |
| T2 Daily Alcohol | -0.008 | 0.014 | 0.557 |  | 0.009 | 0.015 | 0.559 |  | **-0.039** | **0.018** | **0.035** |  | -0.012 | 0.014 | 0.405 |
| T2 Smoker | **0.033** | **0.014** | **0.016** |  | 0.012 | 0.015 | 0.403 |  | 0.008 | 0.019 | 0.646 |  | 0.005 | 0.014 | 0.729 |
| T2 BMI | — | — | — |  | **0.088** | **0.015** | **<0.001** |  | **-0.099** | **0.018** | **<0.001** |  | **0.144** | **0.016** | **<0.001** |
|  |  |  |  |  |  |  |  |  |  |  |  |  |  |  |  |
| **Time 3 – WCT** |  |  |  |  |  |  |  |  |  |  |  |  |  |  |  |
| Age | 0.006 | 0.013 | 0.64 |  | **-0.03** | **0.011** | **0.007** |  | **-0.024** | **0.012** | **0.05** |  | **-0.042** | **0.012** | **0.001** |
| Male Gender | **-0.091** | **0.015** | **<0.001** |  | **-0.088** | **0.015** | **<0.001** |  | **-0.1** | **0.015** | **<0.001** |  | **-0.099** | **0.015** | **<0.001** |
| TDI | 0.023 | 0.015 | 0.129 |  | 0.018 | 0.015 | 0.218 |  | 0.025 | 0.015 | 0.096 |  | 0.011 | 0.016 | 0.498 |
| Disease | -0.028 | 0.016 | 0.085 |  | -0.028 | 0.015 | 0.066 |  | -0.023 | 0.015 | 0.13 |  | -0.021 | 0.015 | 0.158 |
| White British | 0.008 | 0.014 | 0.556 |  | **-0.03** | **0.014** | **0.039** |  | -0.027 | 0.015 | 0.072 |  | -0.027 | 0.014 | 0.063 |
| Retired (retired) | **0.034** | **0.014** | **0.017** |  | **0.046** | **0.016** | **0.003** |  | **-0.045** | **0.016** | **0.005** |  | **-0.038** | **0.016** | **0.017** |
| Living alone | -0.011 | 0.013 | 0.379 |  | 0.010 | 0.014 | 0.497 |  | 0.011 | 0.014 | 0.455 |  | — | — | — |
| Social network | **0.026** | **0.012** | **0.030** |  | **-0.037** | **0.014** | **0.012** |  | **-0.035** | **0.014** | **0.016** |  | — | — | — |
| XF0 (baseline psych) | 0.013 | 0.017 | 0.448 |  | — | — | — |  | 0.020 | 0.015 | 0.189 |  | -0.013 | 0.018 | 0.462 |
| T2 Daily Alcohol | -0.023 | 0.014 | 0.099 |  | -0.024 | 0.014 | 0.094 |  | -0.025 | 0.014 | 0.085 |  | -0.022 | 0.014 | 0.123 |
| T2 Smoker | 0.026 | 0.014 | 0.066 |  | 0.023 | 0.014 | 0.101 |  | 0.025 | 0.014 | 0.082 |  | 0.021 | 0.014 | 0.143 |
| T2 BMI | — | — | — |  | **0.084** | **0.013** | **<0.001** |  | **0.089** | **0.013** | **<0.001** |  | **0.074** | **0.014** | **<0.001** |

*Note.* β = standardized regression coefficient; SE = robust standard error; TDI = Townsend Deprivation Index; BMI = body mass index; WCT = weekly mobile phone call time; XF0 = baseline psychological frailty. Bold rows indicate covariates added in the current analysis (employment/retirement, living alone, social network, and baseline psychological frailty). Dashes (—) indicate the covariate was not included in that model: BMI was not included in physical frailty models (it is a component of the physical frailty score); living alone and social network were not included in social frailty models (they are components of the social frailty score); XF0 was not included in the psychological frailty model (it is the baseline of the same construct). “White British” is the reference category for ethnicity. T1 = baseline assessment (Instance 0, 2006-2010); T2 = first follow-up (Instance 2, 2014 onwards); T3 = second follow-up (Instance 3, 2019 onwards). All estimates are from the unconstrained RI-CLPMs with MLR estimation and FIML handling of missing data.

**Supplementary Table 7**

*Model Fit Indices for RI-CLPMs of Weekly mobile phone call time and Frailty Dimensions*

| **Fit index** | **Physical Frailty** | |  | **Psychological Frailty** | |  | | | **Cognitive Frailty** | |  | | | **Social Frailty** | |
| --- | --- | --- | --- | --- | --- | --- | --- | --- | --- | --- | --- | --- | --- | --- | --- |
|  | **Constrained** | **Unconstrained** |  | **Constrained** | **Unconstrained** | |  | **Constrained** | | **Unconstrained** | |  | **Constrained** | | **Unconstrained** |
| **χ²** | 520.99 | 341.71 |  | 728.63 | 454.43 | |  | 508.97 | | 410.71 | |  | 841.38 | | 383.89 |
| **df** | 63 | 55 |  | 75 | 67 | |  | 75 | | 67 | |  | 51 | | 43 |
| ***P*** | <0.001 | <0.001 |  | <0.001 | <0.001 | |  | <0.001 | | <0.001 | |  | <0.001 | | <0.001 |
| **CFI** | 0.954 | 0.971 |  | 0.946 | 0.968 | |  | 0.940 | | 0.952 | |  | 0.925 | | 0.968 |
| **TLI** | 0.905 | 0.932 |  | 0.898 | 0.933 | |  | 0.882 | | 0.896 | |  | 0.836 | | 0.916 |
| **RMSEA** | 0.037 | 0.031 |  | 0.041 | 0.034 | |  | 0.034 | | 0.032 | |  | 0.055 | | 0.039 |
| **SRMR** | 0.018 | 0.016 |  | 0.022 | 0.021 | |  | 0.018 | | 0.017 | |  | 0.028 | | 0.026 |
| **AIC** | 22594.8 | 22431.5 |  | 24519.9 | 24261.7 | |  | 56415.8 | | 56333.6 | |  | 15387.5 | | 14946.0 |
| **BIC** | 23109.7 | 22999.2 |  | 25029.6 | 24823.7 | |  | 56964.7 | | 56934.8 | |  | 15858.6 | | 15469.5 |

*Model comparisons (unconstrained vs. constrained)*

| **Comparison** | **Physical frailty** | **Psychological frailty** | **Cognitive frailty** | **Social frailty** |
| --- | --- | --- | --- | --- |
| **Δχ² (Δdf = 8)** | 190.22 | 227.73 | 87.57 | 398.43 |
| ***P*** | <0.001 | <0.001 | <0.001 | <0.001 |
| **ΔBIC** | -110.5 | -205.9 | -29.9 | -389.1 |

*Note.* All chi-square difference tests were significant (*P* < 0.001), indicating that the unconstrained model (allowing path coefficients to vary over time) fitted significantly better than the constrained model for each frailty dimension. Lower AIC and BIC values also favour the unconstrained model. Fit indices are based on robust maximum likelihood (MLR) estimation with full information maximum likelihood (FIML) handling of missing data. T1, T2, T3 correspond to the three assessment waves (2006–2010, 2014+, 2019+), respectively.

**Supplementary Table 8**

*Comparison of Baseline Characteristics Between Included and Excluded Participants*

| **Variable** | **Included (N=5,533)** | **Excluded (N=259)** | **Difference** | ***P*** | **Test** |
| --- | --- | --- | --- | --- | --- |
| **Age (years), Mean ± SD** | 52.50 ± 7.22 | 54.82 ± 7.37 | -2.32 | **< 0.001** | t-test |
| **Sex (Male), n (%)** | 2701 (48.8%) | 101 (39.0%) | +9.8% | **0.002** | Chi-square |
| **TDI, Mean ± SD** | -1.92 ± 2.65 | -1.71 ± 2.86 | -0.22 | 0.235 | t-test |
| **White British, n (%)** | 5117 (92.5%) | 234 (90.3%) | +2.1% | **0.018** | Chi-square |
| **Current smoker, n (%)** | 320 (5.8%) | 17 (6.6%) | -0.8% | 0.741 | Chi-square |
| **Daily alcohol use, n (%)** | 1138 (20.6%) | 58 (22.4%) | -1.8% | 0.447 | Chi-square |
| **BMI (kg/m²), Mean ± SD** | 26.59 ± 4.10 | 26.53 ± 4.51 | +0.06 | 0.844 | t-test |
| **Unemployed/Retired, n (%)** | 1136 (20.6%) | 71 (27.8%) | -7.2% | **0.007** | Chi-square |
| **Living alone, n (%)** | 777 (14.0%) | 36 (13.9%) | +0.1% | 0.062 | Chi-square |
| **Social network score, Mean ± SD** | 0.35 ± 0.21 | 0.35 ± 0.21 | -0.00 | 0.718 | t-test |

*Note.*  Included participants (n = 5,533) were those who had valid weekly mobile phone call time (WCT) data at all three assessments (T1, T2, and T3) and had non-missing data for all items across the four frailty dimensions at least one time point. Excluded participants (n = 259) were those who had valid WCT data at all three assessments but were excluded because at least one item across the four frailty dimensions was missing at all three time points. All characteristics were measured at baseline (T1). Data are presented as Mean ± SD for continuous variables and n (%) for categorical variables. P-values were derived from independent two-sample t-tests (continuous variables) and chi-square tests (categorical variables). The significant differences in age, sex, and socioeconomic indicators suggest a healthy-volunteer selection bias, which may limit generalizability.

**Supplementary Table 9 - Sensitivity Analysis: RI-CLPM Model Fit Indices and Cross-Lagged Paths Using Largest Available Samples and WCT Coded as Minutes per Week**

*Part A. Model Fit Indices and Model Comparison*

| **Fit index** | **Physical Frailty** | |  | **Psychological Frailty** | |  | | | **Cognitive Frailty** | |  | | | **Social Frailty** | |
| --- | --- | --- | --- | --- | --- | --- | --- | --- | --- | --- | --- | --- | --- | --- | --- |
|  | **Constrained** | **Unconstrained** |  | **Constrained** | **Unconstrained** | |  | **Constrained** | | **Unconstrained** | |  | **Constrained** | | **Unconstrained** |
| **χ²** | 414.99 | 265.70 |  | 664.92 | 431.08 | |  | 423.60 | | 365.55 | |  | 843.46 | | 398.41 |
| **df** | 51 | 43 |  | 63 | 55 | |  | 63 | | 55 | |  | 51 | | 43 |
| ***P*** | <0.001 | <0.001 |  | <0.001 | <0.001 | |  | <0.001 | | <0.001 | |  | <0.001 | | <0.001 |
| **CFI** | 0.960 | 0.976 |  | 0.948 | 0.968 | |  | 0.945 | | 0.952 | |  | 0.921 | | 0.965 |
| **TLI** | 0.913 | 0.937 |  | 0.899 | 0.928 | |  | 0.887 | | 0.888 | |  | 0.829 | | 0.909 |
| **RMSEA** | 0.036 | 0.030 |  | 0.042 | 0.036 | |  | 0.033 | | 0.033 | |  | 0.054 | | 0.039 |
| **SRMR** | 0.017 | 0.015 |  | 0.024 | 0.023 | |  | 0.018 | | 0.018 | |  | 0.027 | | 0.025 |
| **AIC** | 24374.8 | 24241.5 |  | 27113.1 | 26895.3 | |  | 59375.3 | | 59333.3 | |  | 17352.8 | | 16923.8 |
| **BIC** | 24852.0 | 24771.7 |  | 27587.3 | 27422.1 | |  | 59887.4 | | 59897.9 | |  | 17827.2 | | 17450.9 |

*Model comparisons (unconstrained vs. constrained)*

| **Comparison** | **Physical frailty** | **Psychological frailty** | **Cognitive frailty** | **Social frailty** |
| --- | --- | --- | --- | --- |
| **Δχ² (Δdf = 8)** | 149.29 | 233.84 | 58.05 | 445.05 |
| ***P*** | <0.001 | <0.001 | <0.001 | <0.001 |
| **ΔBIC** | +80.3 | +165.2 | -10.5 | +376.3 |

*Note to Part A:* Sensitivity analyses used (1) each frailty dimension's largest available sample instead of the four‑way intersection (PF: n = 5,647; XF: n = 5,792; CF: n = 5,671; SF: n = 5,792), and (2) WCT recoded as category midpoints in minutes per week (2.5, 17, 45, 120, 300, 420) instead of the ordinal 1‑6 scale. ΔBIC = BIC_constrained − BIC_unconstrained; negative values favour the constrained model. The unconstrained model was preferred for all dimensions based on significant Δχ² tests (all P < 0.001). For CF, ΔBIC marginally favoured the constrained model (−10.5), but the unconstrained model was retained to maintain consistency with the main analysis. All models were estimated with MLR and FIML. PF = physical frailty; XF = psychological frailty; CF = cognitive frailty; SF = social frailty; WCT = weekly mobile phone call time. T1, T2, T3 correspond to the three assessment waves (2006–2010, 2014+, 2019+).

*Part B. Cross-Lagged Paths*

| **Path** | **Physical frailty**  **(n=5,647)** | **Psychological frailty**  **(n=5,792)** | **Cognitive frailty**  **(n=5,671)** | **Social frailty**  **(n=5,792)** |
| --- | --- | --- | --- | --- |
| **WCT T1 → Frailty T2** |  |  |  |  |
| *β (SE)* | 0.039 (0.027) | 0.015 (0.022) | -0.006 (0.026) | 0.003 (0.024) |
| *95% CI* | [-0.014, 0.092] | [-0.028, 0.058] | [-0.057, 0.045] | [-0.044, 0.050] |
| *P / P_FDR* | 0.152 / 0.246 | 0.484 / 0.596 | 0.825 / 0.880 | 0.913 / 0.913 |
| **WCT T2 → Frailty T3** |  |  |  |  |
| *β (SE)* | 0.040 (0.017) | 0.054 (0.026) | -0.019 (0.026) | 0.033 (0.019) |
| *95% CI* | [0.006, 0.074] | [0.002, 0.106] | [-0.070, 0.032] | [-0.004, 0.070] |
| *P / P_FDR* | 0.021 / 0.124 | 0.041 / 0.124 | 0.467 / 0.596 | 0.081 / 0.185 |
| **Frailty T1 → WCT T2** |  |  |  |  |
| *β (SE)* | 0.065 (0.046) | 0.064 (0.032) | -0.036 (0.041) | 0.050 (0.030) |
| *95% CI* | [-0.024, 0.154] | [0.001, 0.127] | [-0.116, 0.044] | [-0.009, 0.109] |
| *P / P_FDR* | 0.154 / 0.246 | 0.044 / 0.124 | 0.384 / 0.559 | 0.099 / 0.198 |
| **Frailty T2 → WCT T3** |  |  |  |  |
| *β (SE)* | 0.058 (0.029) | 0.067 (0.025) | -0.009 (0.029) | 0.058 (0.026) |
| *95% CI* | [0.001, 0.115] | [0.018, 0.116] | [-0.066, 0.048] | [0.007, 0.109] |
| *P / P_FDR* | 0.047 / 0.124 | 0.007 / 0.109 | 0.749 / 0.856 | 0.024 / 0.124 |

*Note to Part B:* *β* = standardized regression coefficient; *SE* = robust standard error; *95% CI* = 95% confidence interval; *P_FDR* = Benjamini‑Hochberg false discovery rate‑adjusted p‑value across all 16 cross‑lagged paths. No cross‑lagged paths remained statistically significant after FDR correction in the sensitivity analysis, though the direction and magnitude of the coefficients were consistent with the main analysis (Table 1). For cognitive frailty, higher scores indicate better cognitive function. PF = physical frailty; XF = psychological frailty; CF = cognitive frailty; SF = social frailty; WCT = weekly mobile phone call time. T1, T2, T3 correspond to the three assessment waves (2006–2010, 2014+, 2019+).

**Supplementary Figure 1**

***Supplementary Figure 1.*** Conceptual random intercept cross-lagged panel model (RI-CLPM) across three time points.

*Note.* The *between* component represent the random intercepts. These capture a unit’s time-invariant deviation from the grand mean and thus represent the stable differences between units (trait like stability). The *within* components are the differences between a unit’s observed measurements (rectangle boxes represent observed scores at each time point) and the unit’s expected score based on the grand mean and its random intercepts. The *auto-regressive effects* (α’s and δ’s) represent the within-person carry-over effects (temporal stability of constructs). The *cross-lagged effects* (β’s and γ’s) represent the spill-over of the state in one domain into the state of another (predicting change). Vertical/curved arrows represent correlations (covariances for both the within and between components in the model).

**Supplementary Figure 2**

*Directed Acyclic Graph (DAG) of the Random Intercept Cross-Lagged Panel Model (RI-CLPM) for Weekly Call Time and Frailty.*

***Supplementary Figure 2.*** This figure illustrates the adjustment relationships between covariates and baseline weekly call time (WCT) and baseline frailty. All covariates were measured at baseline (T1). Covariate inclusion varied by model: XF0 (baseline psychological frailty) was not adjusted for in the psychological frailty model; BMI was not adjusted for in the physical frailty model (as it is a component indicator of the physical frailty score); living alone and social network were not adjusted for in the social frailty model (as they are component indicators of the social frailty score). Physical activity and sleep disturbance were included as time-invariant covariates. All other covariates were included in the models for each dimension. For details, see Methods. Given the observational design of this study, these associations should not be interpreted as causal.
